# Supplementary material for: Barriers to implementation of domestic violence prevention policies and programs in northwestern Ethiopia: A qualitative implementation research
Source: PLOS Glob Public Health. 2025 Mar 18;5(3):e0004266. doi: 10.1371/journal.pgph.0004266 (PMC11918427; doi:10.1371/journal.pgph.0004266)
Supplement: S1 Checklist — COREQ Reporting Checklist. (DOCX) [file pgph.0004266.s001.docx]

**COREQ Reporting Checklist**

The table below provides a complete description of COREQ checklist items as they appear in the manuscript.

| **No. Item** | **Guide questions/description** | **Page Checklist Items are Reported in Manuscript** |
| --- | --- | --- |
| **Domain 1:  Research team and reﬂexivity** | |  |
| ***Personal Characteristics*** |  |  |
| 1. Interviewer/facilitator | Which author/s conducted the interview or focus group? | Page #9 (Line 197) |
| 2. Credentials | What were the researcher’s credentials? E.g. PhD, MD | Page #9 (Line 197) |
| 3. Occupation | What was their occupation at the time of the study? | Page #10 (Line 213-214) |
| 4. Gender | Was the researcher male or female? | Males, **Page #9** (Line 197) |
| 5. Experience and training | What experience or training did the researcher have? | Page #9 (Line 200-201) |
| *Relationship with participants* | |  |
| 6. Relationship established | Was a relationship established prior to study commencement? | Page #5&6 (Lines 121-122). |
| 7. Participant knowledge of the interviewer | What did the participants know about the researcher? e.g. personal goals, reasons for doing the research | Page #5&6: The researchers initially introduced themsleves briefly before the commencement of the interview. |
| 8. Interviewer characteristics | What characteristics were reported about the interviewer/facilitator? e.g. Bias, assumptions, reasons and interests in the research topic | Page #9 (Lines 209-211) |
| **Domain 2:**  **Study Design and *Theoretical Framework*** | |  |
| 9. Methodological orientation and Theory | What methodological orientation was stated to underpin the study? e.g. grounded theory, discourse analysis, ethnography, phenomenology, content analysis | The study was guided by the Consolidated Framework for Implementation Research (CFIR) and content analysis **Page #6: (Lines 132-137).** |
| ***Participant selection*** |  |  |
| 10. Sampling | How were participants selected? e.g. purposive, convenience, consecutive, snowball | **Page #7 (Lines 160-161):** A purposive sampling technique was used. |
| 11. Method of approach | How were participants approached? e.g. face-to-face, telephone, mail, email | **Page # 8:** All interviews were conducted face-to-face (Lines 175-195). |
| 12. Sample size | How many participants were in the study? | 43 participants took part in the study, **Page #7(Line 160)** |
| 13. Non-participation | How many people refused to participate or dropped out? Reasons? | Page 7, participation was voluntary and no one refused. |
| ***Setting*** |  |  |
| 14. Setting of data collection | Where was the data collected? e.g. home, clinic, workplace | **Pages #8 & 9**: office for KII, home and village for IDIs and FGDs (Lines 190-192). |
| 15. Presence of non-participants | Was anyone else present besides the participants and researchers? | **Page #5 & 8**: No one was around during the interviews. |
| 16. Description of sample | What are the important characteristics of the sample? e.g. demographic data, date | **Page # 11,** Table 1 provides information about the background characteristics of the participants. |
| ***Data collection*** |  |  |
| 17. Interview guide | Were questions, prompts, guides provided by the authors? Was it pilot tested? | **Page #8:** semi-structure interview guides (Lines 177-178). |
| 18. Repeat interviews | Were repeat interviews carried out? If yes, how many? | No, interview was conducted once per participant |
| 19. Audio/visual recording | Did the research use audio or visual recording to collect the data? | **Pages 8,9, &10**: Interviews were audio-record (Lines 175-195). |
| 20. Field notes | Were ﬁeld notes made during and/or after the interview or focus group? | **Pages 8,9,&10:** field notes were taken during both for interview and FGDs |
| 21. Duration | What was the duration of the interviews or focus group? | **Page 8-9**: 30-45 minutes for KII and IDI, and up to 90 minutes for FGDs (Lines 185 & 195) |
| 22. Data saturation | Was data saturation discussed? | **Page #7:** sample size was determined by information saturation (Lines 160-161). |
| 23. Transcripts returned | Were transcripts returned to participants for comment and/or correction? | findings were validated instead of each transcription |
| **Domain 3:  *Data* Analysis and Findings** | |  |
| 24. Number of data coders | How many data coders coded the data? | Two coders, **Page # 10**. |
| 25. Description of the coding tree | Did authors provide a description of the coding tree? | Yes, a description of the coding process is provided on **page 10 (Lines 219-230).** |
| 26. Derivation of themes | Were themes identiﬁed in advance or derived from the data? | Some themes were identified in advance based on the consolidated framework of implementation research while new themes were generated through the analysis (**Page # 10: lines 220-229)** |
| 27. Software | What software, if applicable, was used to manage the data? | Yes, NVivo 11, see **page #10 (line 220).** |
| 28. Participant checking | Did participants provide feedback on the ﬁndings? | Yes, during the result validation workshop, **Page #9 (Lines 211-213).** |
| ***Reporting*** |  |  |
| 29. Quotations presented | Were participant quotations presented to illustrate the themes/ﬁndings? Was each quotation identiﬁed? e.g. participant number | Yes, see **Pages 12, 13, 14, & 15.** |
| 30. Data and ﬁndings consistent | Was there consistency between the data presented and the ﬁndings? | Yes, there is consistency between the data and findings throughout the results section **Pages 12, 13, 14 and 15.** |
| 31. Clarity of major themes | Were major themes clearly presented in the ﬁndings? | **Yes, see Pages 12, 13, 14 and 15.** |
| 32. Clarity of minor themes | Is there a description of diverse cases or discussion of minor themes? | **Yes, see pages 12-18.** |
