# Supplementary material for: Barriers to implementation of domestic violence prevention policies and programs in northwestern Ethiopia: A qualitative implementation research
Source: PLOS Glob Public Health. 2025 Mar 18;5(3):e0004266. doi: 10.1371/journal.pgph.0004266 (PMC11918427; doi:10.1371/journal.pgph.0004266)
Supplement: Attachment — Submitted filename: Response_to_Reviewers_auresp_2.docx [file pgph.0004266.s004.docx]

Department of Population, Family and Reproductive Health,

University of Ghana School of Public Health
P. O. Box LG 13
Legon, Accra
Ghana
[amanu@ug.edu.gh](mailto:amanu@ug.edu.gh)

26 December 2024

The Editor
PLOS Global Public Health

Dear Editor,

**RESPONSE TO REVIEWERS
 MANUSCRIPT: PGPH-D-24-00970**

I wish to submit our revised manuscript titled **“Barriers to implementation of domestic violence prevention policies and programs in northwestern Ethiopia: Qualitative implementation research”** for publication in PLOS Global Public Health.

We have responded appropriately to the reviewer’s comments detailing the various pages where the COREQ checklist items have been reported in the manuscript. Additionally, we have thoroughly reviewed the manuscript to sort out all the grammar errors.

**Kindly find below our response to the specific comments:**

**Comment 1:**

Please use the COREQ checklist, or another relevant reporting checklist, to ensure complete reporting of the methods and results. A supplementary document should be included detailing where in the manuscript the checklist items are reported.

**Response 1:**

We have carefully reviewed the manuscript and have responded to the 32-item checklist as follows:

| **No. Item** | **Guide questions/description** | **Page Checklist Items are Reported in Manuscript** |
| --- | --- | --- |
| **Domain 1:  Research team and reﬂexivity** | |  |
| ***Personal Characteristics*** |  |  |
| 1. Interviewer/facilitator | Which author/s conducted the interview or focus group? | Page #9 (Line 197) |
| 2. Credentials | What were the researcher’s credentials? E.g. PhD, MD | Page #9 (Line 197) |
| 3. Occupation | What was their occupation at the time of the study? | Page #10 (Line 213-214) |
| 4. Gender | Was the researcher male or female? | Males, **Page #9** (Line 197) |
| 5. Experience and training | What experience or training did the researcher have? | Page #9 (Line 200-201) |
| *Relationship with participants* | |  |
| 6. Relationship established | Was a relationship established prior to study commencement? | Page #5&6 (Lines 121-122). |
| 7. Participant knowledge of the interviewer | What did the participants know about the researcher? e.g. personal goals, reasons for doing the research | Page #5&6: The researchers initially introduced themsleves briefly before commentcment of the interview. |
| 8. Interviewer characteristics | What characteristics were reported about the interviewer/facilitator? e.g. Bias, assumptions, reasons and interests in the research topic | Page #9 (Lines 209-211) |
| **Domain 2:**  **Study Design and *Theoretical Framework*** | |  |
| 9. Methodological orientation and Theory | What methodological orientation was stated to underpin the study? e.g. grounded theory, discourse analysis, ethnography, phenomenology, content analysis | The study was guided by the Consolidated Framework for Implementation Research (CFIR) and content analysis **Page #6: (Lines 132-137).** |
| ***Participant selection*** |  |  |
| 10. Sampling | How were participants selected? e.g. purposive, convenience, consecutive, snowball | **Page #7 (Lines 160-161):**  A purposive sampling technique was used. |
| 11. Method of approach | How were participants approached? e.g. face-to-face, telephone, mail, email | **Page # 8:** All interviews were conducted face-to-face (Lines 175-195). |
| 12. Sample size | How many participants were in the study? | 43 participants took part in the study, **Page #7(Line 160)** |
| 13. Non-participation | How many people refused to participate or dropped out? Reasons? | Page 7, participation was voluntary and no one refused. |
| ***Setting*** |  |  |
| 14. Setting of data collection | Where was the data collected? e.g. home, clinic, workplace | **Pages #8 & 9**: office for KII, home and village for IDIs and FGDs (Lines 190-192). |
| 15. Presence of non-participants | Was anyone else present besides the participants and researchers? | **Page #5 & 8**: No one was around during the interviews. |
| 16. Description of sample | What are the important characteristics of the sample? e.g. demographic data, date | **Page # 11,** Table 1 provides information about the background characteristics of the participants. |
| ***Data collection*** |  |  |
| 17. Interview guide | Were questions, prompts, guides provided by the authors? Was it pilot tested? | **Page #8:** semi-structure interview guides (Lines 177-178). |
| 18. Repeat interviews | Were repeat interviews carried out? If yes, how many? | No, interview was conducted once per participant |
| 19. Audio/visual recording | Did the research use audio or visual recording to collect the data? | **Pages 8,9, &10**: Interviews were audio-record (Lines 175-195). |
| 20. Field notes | Were ﬁeld notes made during and/or after the interview or focus group? | **Pages 8,9,&10:** field notes were taken during both for interview and FGDs |
| 21. Duration | What was the duration of the interviews or focus group? | **Page 8-9**: 30-45 minutes for KII and IDI, and up to 90 minutes for FGDs (Lines 185 & 195) |
| 22. Data saturation | Was data saturation discussed? | **Page #7:** sample size was determined by information saturation (Lines 160-161). |
| 23. Transcripts returned | Were transcripts returned to participants for comment and/or correction? | findings were validated instead of each transcription |
| **Domain 3:  *Data* Analysis and Findings** | |  |
| 24. Number of data coders | How many data coders coded the data? | Two coders, **Page # 10**. |
| 25. Description of the coding tree | Did authors provide a description of the coding tree? | Yes, description of the coding process is provided on **page 10 (Lines 219-230).** |
| 26. Derivation of themes | Were themes identiﬁed in advance or derived from the data? | Some themes were identified in advance based on the consolidated framework of implementation research while new themes were generated through the analysis (**Page # 10: lines 220-229)** |
| 27. Software | What software, if applicable, was used to manage the data? | Yes, NVivo 11, see **page #10 (line 220).** |
| 28. Participant checking | Did participants provide feedback on the ﬁndings? | Yes, during the result validation workshop, **Page #9 (Lines 211-213).** |
| ***Reporting*** |  |  |
| 29. Quotations presented | Were participant quotations presented to illustrate the themes/ﬁndings? Was each quotation identiﬁed? e.g. participant number | Yes, see **Pages 12, 13, 14, & 15.** |
| 30. Data and ﬁndings consistent | Was there consistency between the data presented and the ﬁndings? | Yes, there is consistency between the data and findings throughout the results section **Pages 12, 13, 14 and 15.** |
| 31. Clarity of major themes | Were major themes clearly presented in the ﬁndings? | **Yes, see Pages 12, 13, 14 and 15.** |
| 32. Clarity of minor themes | Is there a description of diverse cases or discussion of minor themes? | **Yes, see pages 12-18.** |

**Comment 2:**

I recommend using a language editing service to identify and correct a few remaining spelling and grammatical errors (e.g., inappropriate uses of the comma in lines 47, 51, 55; "resulting" in line 57 should be "result"; etc.).

**Response 2:**
We have thoroughly reviewed the manuscript and corrected all the grammar errors. The inappropriate commas on Lines 47, 51, and 55 have been corrected, while “resulting” has been changed to **results** (Line 57). In addition, we used the Grammarly grammar checker to correct all the grammatical errors.

We are hopeful that you may find our responses acceptable and approve our manuscript for publication in Plos Global Public Health.

Thank you.

Adom Manu
