# Supplementary material for: Barriers to implementation of domestic violence prevention policies and programs in northwestern Ethiopia: A qualitative implementation research
Source: PLOS Glob Public Health. 2025 Mar 18;5(3):e0004266. doi: 10.1371/journal.pgph.0004266 (PMC11918427; doi:10.1371/journal.pgph.0004266)
Supplement: Attachment — Submitted filename: Response to Reviewers.docx [file pgph.0004266.s003.docx]

**Point by point response to comments**

**Manuscript Title**: Barriers to implementation of domestic violence prevention policies and programs in northwestern Ethiopia: A qualitative implementation research
**Journal:** PLOS Global Public Health

**Manuscript ID**: GPH-D-24-00970

| Section # | Reviewers comment | *Authors response* |
| --- | --- | --- |
| Declaration | We note that your Data Availability Statement is currently as follows: This was a qualitative study. Relevant data that support the findings of this study are presented within the content of the manuscript.    For example, authors should submit the following data:   - The values behind the means, standard deviations and other measures reported; | *Thank you for the comment. The basic profile of qualitative study participants.* |
|  | Reviewer #1: |  |
| General comment | The study employs a qualitative approach to investigate the challenges and barriers in implementing domestic violence prevention policies and programs in northwestern Ethiopia. Using the Consolidated Framework for Implementation Research (CFIR), the researchers conducted in-depth interviews, key informant interviews, and focus group discussions with purposively selected participants involved in policy implementation. The study utilized an established qualitative data analysis method, including thematic analysis using NVivo 11 software. The findings reveal multiple barriers across various domains, including evidence-based intervention, inner setting, outer setting, individual behaviors, and process-related factors. The results are presented clearly, supported by direct quotes from participants, and organized under thematic subheadings. This research provides valuable insights into the complexities of implementing domestic violence prevention strategies in the region and can inform future efforts to improve policy implementation and program effectiveness. Overall, the manuscript is technically sound. I will present my comments by sections. | *Many thanks for the constructive comment.* |
| 1. Introduction: | The introduction provides a comprehensive background on the prevalence and impact of violence against women (VAW) i) globally, ii) in Ethiopia and iii) the legal and policy framework in Ethiopia. It highlights the significance by citing statistics and referencing international declarations and local laws aimed at combating VAW, thus establishing the relevance of the study in the context of public health and human rights. | *Many thanks for the constructive comment.* |
| 1.1. Line 56: | "Domestic VAW is a manifestation of inequality and power imbalance in relationship" should be "in relationships." | *Corrected (see Line 75 of trach change version)* |
| 1.2. Line 66: | Lifetime prevalence of VAW varies from one part of the country to another – Varies how much? Any references? | *Many thanks for the comment. Relevant references are cited. See track change.* |
| 1.3. Line 88: | “Setting the minimum age of marriage at 18 year among” it should be “years”. | *Corrected as per suggestion. See track changes Line 97.* |
| 1.4. Line 104: | "…. in Ethiopia that seriously undermines women and girls’ health and social status" should be "women's and girls’ health and social status.” | *This has been corrected (see Line 114 of track changes).* |
| 2. Methods | The methods section is appropriate for the study's objectives. It provides a clear and detailed description of the study design, setting, sampling method, data collection procedures, data management and analysis, and ethical considerations. The use of the Consolidated Framework for Implementation Research (CFIR) is well-justified and aligns with the study's aim to identify barriers to the implementation of domestic violence prevention policies and programs. | *Many thanks for the constructive comment.* |
| 2.1. | A few suggestions: The methods section does not discuss the researchers' potential biases or reflexivity, which is important in qualitative research to acknowledge and mitigate any influence on data collection and analysis. | *Thanks for this suggestion. We have included authors’ reflexivity statement (Lines 204-217)* |
| 2.2. | While the section mentions the use of IDIs, KIIs, and FGDs, it could benefit from more specific details on the interview guides' content and how they were developed. | *Many thanks, for the comment. We have done systematic review and also tool validation for the formative assessment. This study is emanated from that mother documents. Mainly focused on VAW, attitudes towards gender* equa**lity, possible prevention measures, and policy implementation** barriers. |
| 2.3. | Sample size. Why 43 participants? I didn´t find any justification for this number | *Many thanks for the comment.th* |
| 2.4. | What about data saturation? The section does not mention whether data saturation was achieved. | *Thanks, the data saturation is no included in the sampling section. See track change* |
| 2.5. | There is no information on the role of researchers during data collection and analysis, which could provide insights into how data were interpreted and any potential biases. Maybe add a section discussing the researchers' potential biases and how they were addressed during the study to enhance the credibility of the findings? Author´s contribution state that only one author conducted the data analysis, which it is not the usual protocol of qualitative data analysis. A single coder can have several implications in the analysis such as, lack of inter-coder reliability and reflexivity (For more information see, for e.g., COREQ protocol). Thus, confirmation bias should be acknowledged as a limitation of the study. | *The Authors involvement is now well addressed in the* ***reflexivity statement****. See track change* |
| 2.6. | Provide more specific information on the content of the interview guides and how they were developed to give readers a clearer understanding of the data collection process. | *Many thanks, for the comment. We conducted a systematic review and tool validation for the formative assessment. This study is emanated from that mother documents. Mainly focused on VAW, attitudes towards gender* equa**lity, possible prevention measures, and policy implementation** barriers. |
| 2.7. Line 187: | Was it then an “hybrid approach” to thematic analysis? | *Yes, it was a hybrid approach using deductive and inductive coding, than thematic areas identified.* |
| 3. Results | The results section is appropriate for the study's objectives. It provides a clear and detailed presentation of the findings, including both quantitative demographics and qualitative data. | *Many thanks for the constructive comment.* |
| 3.1 | There is some repetition in the quotes and points made, particularly regarding the lack of political will and poor collaboration. Reduce repetition in the quotes and points made to streamline the content and improve clarity. For instance, political will and political commitment are closely related concepts, it would be useful to have a table with the description, focus and nature of the themes included in the results section. | *Many thanks for the comment, we have revised align similar concepts. See track changes* |
| 3.2 | The results present the KII results and FGDs in a mixed analysis, it would bring some clarity if the FGDs were classified in the citations such as, FGD1; FGD2, etc. It clearly help attribute quotes and findings to their respective sources and see which FGDs were more cited, for instance. | *Many thanks for the constructive comment.* |
| 3.3 Line 288: | punctuation | *Thanks, we have addressed the concern accordingly* |
| 4. Discussion | The discussion provides a thorough examination of the barriers to implementing domestic violence against women (VAW) prevention policies in northwestern Ethiopia. | *Many thanks for the constructive comment.* |
| 4.1. | While barriers are well-identified, the discussion could benefit from more in-depth exploration of potential solutions. | *Many thanks for the constructive comment.* |
| 4.2 | The discussion relies heavily on qualitative findings. Incorporating some quantitative data could strengthen the arguments. | *Many thanks for the constructive comment.* |
| 4.3. | Some ideas, such as the need for intersectoral collaboration, are repeated multiple times without adding new insights. E.g. intersectoral collaboration (IC) was mentioned in sentences starting at line 348, 360, 372, 380. To avoid repetition and enhance clarity, this point can be consolidated into the same section. Or, if the goal of the authors is to indeed focus the discussion on IC, maybe divide it by parts such as, Role and importance of IC; Barriers to effective IC; Strategies for Improving IC. (This is clearly just a suggestion for authors consideration) | *Many thanks for the comment and we have tried to reduce repetitions and addressed the concerns accordingly. See track change* |
| 5. Strenghts and Limitations | 5.1. Line 395-396: "This study used mixed qualitative methods to explore implementation barriers that are recommended for the findings' transferability." • This sentence is a bit awkward. It could be rephrased for clarity, though it's not strictly a typo. | *Many thanks for the comment and we have rephrased to improve readability of the manuscript.* |
| 6. Conclusion: | 6.1 Line 410: "...will be super helpful in reducing domestic violence in northeastern Ethiopia." instead of super, perhaps use "highly" or "extremely", a bit more formal. • Northeastern or Northwestern?! | *Many thanks for the comment. Corrected as “extremely” and “northwestern” (Line 476).* |
