# Supplementary material for: Barriers to implementation of domestic violence prevention policies and programs in northwestern Ethiopia: A qualitative implementation research
Source: PLOS Glob Public Health. 2025 Mar 18;5(3):e0004266. doi: 10.1371/journal.pgph.0004266 (PMC11918427; doi:10.1371/journal.pgph.0004266)
Supplement: Attachment — Submitted filename: Response_to_Reviewers_auresp_3.docx [file pgph.0004266.s005.docx]

**Response to Reviewer Comments**

**Manuscript Title**: Barriers to implementation of domestic violence prevention policies and programs in northwestern Ethiopia: A qualitative implementation research

**Journal:** PLOS Global Public Health

**Manuscript ID**: PGPH-D-24-00970

**Comment:** Please upload the completed COREQ reporting checklist as a supplementary file alongside your revised manuscript.

**Response:** We have provided a supplementary file detailing the various pages where the COREQ checklist items have been reported in the manuscript.

Thank you.
